# *Hap2*, a novel gene in *Babesia bigemina* is expressed in tick stages, and specific antibodies block zygote formation

**DOI:** 10.1186/s13071-017-2510-0

**Published:** 2017-11-13

**Authors:** Minerva Camacho-Nuez, Diego Josimar Hernández-Silva, Elizabeth Jacqueline Castañeda-Ortiz, María Elena Paredes-Martínez, Marisol Karina Rocha-Martínez, María Elizbeth Alvarez-Sánchez, Ricardo Francisco Mercado-Curiel, Gabriela Aguilar-Tipacamu, Juan Mosqueda

**Affiliations:** 1 Posgrado en Ciencias Genómicas, Universidad Autónoma de la Ciudad de México, San Lorenzo, esquina Roberto Gayol, Colonia del Valle Sur, Delegación Benito Juárez, C.P. 03100, Mexico D.F, Mexico; 2 Facultad de Ciencias Naturales, Universidad Autónoma de Querétaro, Av. de las Ciencias s/n Col Juriquilla, C.P, 76230 Queretaro, Mexico; 3 Facultad de Medicina, Universidad Autónoma de Querétaro, Fraccionamiento Prados de la Capilla, Querétaro, Mexico

**Keywords:** Bovine babesiosis, *Babesia bigemina*, HAP2, Gamete fusion

## Abstract

**Background:**

Bovine babesiosis is a tick-borne disease caused by the protozoan parasites of the genus *Babesia*. In their host vector, *Babesia* spp. undergo sexual reproduction. Therefore, the development of sexual stages and the subsequent formation of the zygote are essential for the parasite to invade the intestinal cells of the vector tick and continue its life-cycle. HAP2/GCS1 is a protein identified in plants, protozoan parasites and other organisms that has an important role during membrane fusion in fertilization processes. The identification and characterization of HAP-2 protein in *Babesia* would be very significant to understand the biology of the parasite and to develop a transmission-blocking vaccine in the future.

**Results:**

To isolate and sequence the *hap2* gene DNA from an infected bovine with *Babesia bigemina* was purified. The *hap2* gene was amplified, cloned and sequenced. The sequences of *hap2* from four geographically different strains showed high conservation at the amino acid level, including the typical structure with a signal peptide and the HAP2/GSC domain. Antisera anti-HAP2 against the conserved extracellular region of the HAP2 amino acid sequence were obtained from rabbits. The expression of *hap2* in the host and vector tissues was analyzed by using semi-quantitative RT-PCR, and the protein was examined by western blot and immunofluorescence. Based on the RT-PCR and WB results, HAP2 is expressed in both, sexual stages induced in vitro*,* and in infected ticks as well. We did not detect any expression in asexual erythrocytic stages of *B. bigemina*, relevantly anti-HAP2 specific antibodies were able to block zygotes formation in vitro.

**Conclusion:**

*Babesia bigemina* HAP2 is expressed only in tick-infecting stages, and specific antibodies block zygote formation. Further studies regarding the function of HAP2 during tick infection may provide new insights into the molecular mechanisms of sexual reproduction of the parasite.

**Electronic supplementary material:**

The online version of this article (10.1186/s13071-017-2510-0) contains supplementary material, which is available to authorized users.

## Background

Babesiosis is a tick-borne disease caused by intraerythrocytic protozoans of the genus *Babesia*, which infect a wide range of domestic animals and occasionally humans. The species affecting cattle are *B. bovis* and *B. bigemina*. Current methods for controlling bovine babesiosis are based on two aspects: one is the control of ticks and the second one is the use of therapeutic chemicals to eliminate the pathogen from the infected animal. Both methods have drawbacks, generating resistance [1–3] and high costs, plus a long withdrawal time associated with residue problems in the food chain [4].

Among the many strategies directed at controlling vector-borne diseases is the advancement of transmission-blocking vaccines (TBVs), which have been developed to interrupt the life-cycle of some protozoan parasites, such as *Plasmodium* spp. These vaccines aim to interfere with and block pathogen development within the vector. These vaccines are based on identifying surface-expressed proteins during the life-cycle stages of parasites inside the vector. In *Plasmodium*, different surface proteins of gametes, such as Pfs48/45 and Pfs230, have proven to be good immunogens [5, 6]. HAP2 protein has also been proposed as a candidate for this type of vaccine [7]. HAP2/GCS1 is a highly conserved protein, expressed in male gametocytes and it was originally identified in *Arabidopsis thaliana* [8, 9] and later in genomes of green algae, flowering plants and *Plasmodium* spp. [10–12]. There is the hypothesis that this protein is an ancient gamete fusogen [13] and it has a very similar overall architecture to class II viral fusion proteins [14, 15]. Different studies of this protein have suggested that it has an important function in fertilization. When the *hap2* gene is absent or mutated, the zygote formation is completely blocked indicating its relevance in this event [9, 16, 17]. In *Plasmodium berguei,* HAP2 is essential for the fusion of gamete surface membranes but not necessary for the adhesion of male and female gametes, and specific antibodies anti-HAP2 block its transmission in vivo and in vitro [17].

*Babesia* parasites have a complex life-cycle, including asexual stages in the bovine host and sexual stages in ticks. The development of sexual stages of *Babesia* spp. and the subsequent formation of the zygote are essential for the parasite to invade the intestinal cells of the vector tick and continue its life-cycle, nevertheless very little is known about the molecular events involved in the sexual reproduction of the parasite and sexual stage proteins. There are a few reports in *Babesia* spp. of sexual stage-specific proteins; two proteins encoded by a six-cysteine (6-Cys) gene family Bbo CysA and B have been found to be expressed during sexual stages in *B. bovis* [18]. Meanwhile, in *B. bigemina* sexual stages, the expression of the family of multidomain adhesion CCp proteins (CCp 1–3) has been demonstrated in vitro [19]. The identification and characterization of HAP-2 protein in *B. bigemina* would be very significant to understand the biology of the parasite and to develop a transmission-blocking vaccine in the future. In this study, we isolated and characterized the *hap2* gene of *B. bigemina*, its expression profile in the host and vector infected cells and we also tested if anti-HAP2 specific antibodies were able to block zygote formation in vitro.

## Methods

### Ticks

A *Babesia*-free colony of *Rhipicephalus microplus* (Media Joya strain) was maintained under laboratory conditions. *Rhipicephalus microplus* larvae hatched from 0.5 g of eggs, were placed on an intact calf and 21 days later replete female ticks were collected. To obtain infected ticks, concurrently, *R. microplus* larvae from 0.5 g of eggs were placed on a splenectomized calf. Fourteen days later, the calf was intravenously inoculated with 5 ml of blood infected with *B. bigemina* (Chiapas strain) previously maintained in liquid nitrogen and 21 days later replete female ticks were collected. Replete female ticks fed on infected or uninfected blood were collected a part of the ticks used to obtain total extracts for RNA and also to obtain their midguts at 0, and 72 h post-repletion as previously reported [20]. To confirm infection, hemolymph smears were examined from 30 females, 72 h post-repletion [21].

### Identification of *hap2 gene* in *B. bigemina* genome

To identify *hap2* in the genome of *B. bigemina*, we used the reported sequence for *hap2* in *Plasmodium berguei* (EU369602) [8] as a probe to search in the database of the Sanger Institute (Cambridge, UK) using the Basic Local Alignment Search Tool (BLAST) (http://www.sanger.ac.uk).

### DNA extraction, amplification and sequencing of *hap2* gene

DNA extraction was performed with a Gentra Puregene kit (Gentra Purogene, Hilden, Germany), according to the supplier’s instructions. The final pellet was resuspended in 100 μl of the hydrating solution and incubated for 5 min at 65 °C, to subsequently be stored at -20 °C until use.

To analyze if *hap2* is conserved among geographically different strains we used DNA from the Chiapas-México, Kayseri-Turkey, Rondonia-Brazil, and Seed-México strains. The *hap2* gene of each strain was amplified by PCR using oligonucleotides hap2F (5′-GAT AAG AAT TCA TGA CGC ATG CCG TGC TGA AC-3′) and hap2R (5′-GAT AAG AAG CTT CTA CAC CTC GTC GCT ATG GC-3′) and Platinum® *Taq* DNA Polymerase High Fidelity (Invitrogen, Carlsbad, USA). The conditions of PCR were as follow: an initial denaturing step at 94 °C for 5 min, followed by 30 cycles at 94 °C for 1 min, 60 °C for 30 s and 72 °C for 2.5 min, with a final extension step at 72 °C for 7 min. The amplicons were cloned into the TOPO TA vector (Invitrogen), according to the manufacturer’s instructions. The inserts of the selected clones (3 of each strain) were sequenced by the dideoxy chain-termination method [22] in an Automatic Sequencer (AB3130 Applied Biosystems). To complete the sequence of the whole gene, two additional intern primers were used: forward hap2IF2 (5′-GTG TGG TGT CAA CGT CAC GG-3′) and reverse hap2IR2 (5′-TGC ACG CTC TGC TCC TCC GA-3′). The sequences were deposited in the GenBank database under the accession numbers: KX768110 (Chiapas-México strain); KX768111 (Kayseri-Turkey strain); KX768112 (Seed-México strain); and KX768113 (Rondonia-Brazil strain).

To deduce the putative introns of the *hap2* gene and the amino acid sequence, the GeneWise tool was used (http://www.ebi.ac.uk/Tools/psa/genewise), considering the amino acid sequence of the putative membrane protein reported in GenBank as a reference (accession number: XM_012913569.1) and nucleotide sequences obtained in this work. The predicted amino acid sequence of *B. bigemina* HAP2 was used to determine the E-values of related proteins by BLAST (http://blast.ncbi.nlm.nih.gov/Blast.cgi). HAP2/GSC1 domain sequence of Chiapas-México strain was aligned with the following sequences: *Babesia bigemina* BBOND strain (XP_012769023.1), *Plasmodium berguei* (EU369602), *Toxoplasma gondii* VAND strain KFH08353.1, *Babesia bovis* strain T2Bo strain (XP_001611806.1) using Clustal Omega (http://www.ebi.ac.uk/Tools/msa/clustalo/).

To identify the signal peptide, a bioinformatics analysis was performed by using the SignalP Server v 4.0 and TMHMM program. 2.0 was used to identify transmembrane helices http://www.cbs.dtu.dk/services/TMHMM/

The alignment of HAP2 amino acid sequences of the different strains was performed with Clustal Omega (http://www.ebi.ac.uk/Tools/msa/clustalo/).

### Induction of sexual stages of *B. bigemina* in vitro

Sexual stages of *B. bigemina* were obtained as described below [23]. Briefly, a splenectomized calf was inoculated with the Chiapas strain of *B. bigemina*, and nine days later blood was obtained. Infected erythrocytes were washed and suspended in a 20% final volume in the induction medium (M199 supplemented with 20% bovine serum, 100 μM xanthurenic acid (Sigma, St. Louis, USA) and antibiotic-antimycotic (Invitrogen) and placed in 75 ml culture flasks (Corning, New York, USA). Cultures were incubated at 28 °C for 18 h. Sexual stages induced in vitro were centrifuged and separated over a 47% percoll gradient. The interface was collected and washed; smears were made and stained with Giemsa to corroborate sexual stages.

### Tissue dissection and protein extraction

The midguts from infected or uninfected ticks of replete females were obtained as we reported previously [20]. Proteins were isolated from midgut using TNTE (50 mM Tris pH 7.4, 150 Mm NaCl, 0.5% Triton and 1 Mm EDTA) as we described before [16].

### Transcription analysis by RT-PCR

RNA from *B. bigemina* infected erythrocytes, induced sexual stages of the parasite, pool of infected and uninfected ticks 0 h and 72 h post-repletion and from the midgut of infected ticks, 72 h post-repletion was purified using TRIzol (Invitrogen). Then, 1 μg of total RNA was reverse-transcribed with ThermoScript™ RT-PCR System for First-Strand cDNA Synthesis (Invitrogen), and used for amplification with Platinum® *Taq* DNA Polymerase High Fidelity (Invitrogen) following the supplier’s instructions. Two μl of cDNA of each sample was used for PCR. The oligonucleotides used for amplification of 375 bp *hap2* were pF (5′-GAC AAA TTC ACC GAC ACG TC-3′) and pR (5′-CTA CAC CTC GTC GCT ATG-3′). Amplification conditions for *hap2* were: an initial denaturing step at 94 °C for 5 min, followed by 30 cycles at 94 °C for 1 min, 58 °C for 30 s and 72 °C for 40 s, with a final extension step at 72 °C for 7 min and 4 °C indefinitely. To obtain a 155 bp amplicon of the *gapdh* as a reference gene, we designed a pair of primers from the sequence BBBOND_0202520: forward (5′-CCG CAC CAT CAA ATT GTA C-3′) and reverse (5′-CGG AGA TGA TGA CCA ACT TG-3′). RNA from uninfected erythrocytes and uninfected ticks was used as negative control.

### Peptide design and antisera production

Based on the multiple alignments of amino acid HAP2 among different strains, we selected the most conserved regions of the extracellular domain to generate two synthetic peptides (pep2 and pep3) of twenty-one and sixteen amino acid residues respectively which were used for rabbit immunization to generate two monospecific polyclonal antisera. The peptides were designed by using the bioinformatics programs ABCPred (http://www.imtech.res.in/raghava/abcpred/), BCEPred (http://www.imtech.res.in/raghava/bcepred/) and IEDB (http://www.iedb.org/). Two peptide sequences with the highest value in all three B cell epitope and antigenic algorithms were selected (pep2: VIISPVRQCIDKGGRSVAEGD, pep3: RKDKPNSGLYIHIQTS, Additional file 1: Figure S1). The peptides were chemically synthesized using a Multiple Antigenic Peptide System with eight asymmetric branches (MAPS 8) (GL Biochem Shanghai Ltd., Shanghai, China). The peptides were suspended in sterile phosphate buffered saline (PBS) to a final concentration of 100 μg per 500 μl of PBS. Each solution was mixed with 500 μl of Montanide ISA 71 VG (Seppic, Paris, France) as an adjuvant, as it was used before [20]. The mixture of each peptide was emulsified and kept at 4 °C until used on the same day. Two, 3-month old New Zealand rabbits were subcutaneously immunized for a total of five times every two weeks. Fifteen days after the last immunization, the rabbits were bled, and the antisera were obtained and stored at -20 °C until used. Additionally, two rabbits were immunised only with the adjuvant and the serum was used as a negative control.

### Indirect immunofluorescence assays

For indirect immunofluorescence assays, *B. bigemina* sexual stages were fixed with 100% methanol, infected and uninfected erythrocytes were fixed with 100% acetone, the slides were left to dry for 20 min at room temperature, and then 20 μl of primary rabbit antibodies against the synthetic peptide 2 (1:80) was added. Pre-immune serum (1:80) was used as a control in each case. Slides were incubated at 37 °C for 30 min, washed three times with PBS-Tween 0.05% for 5 min and then allowed to dry at 37 °C for 15 min. Thereafter, the slides were incubated with 20 μl of Alexa-488 conjugated anti-IgG rabbit antibody (Jackson ImmunoResearch, West Grove, USA) diluted 1:200 in PBS-Tween 0.05% for 1 h, washed 3 times with PBS-Tween 0.05% for 15 min and left to dry. The slides were mounted with glycerol phosphate (1:10) and visualized under a fluorescence microscope (Leica Microsystem, Wetzlar, Germany) with a 100× objective and a specific filter to the spectrum of the Alexa-488 fluorochrome.

### Expression analysis by western blotting (WB)

One μg of protein extracts from uninfected and infected erythrocytes, sexual stages, infected and uninfected tick midguts were separated on 10% SDS-polyacrylamide gels and then electrotransferred onto nitrocellulose membranes (Bio-Rad, Hercules, USA). Non-specific binding was blocked by incubating the membranes overnight in 5% non-fat dried milk in TBS containing 0.05% Tween-20% (TBS-T). The membranes were then washed and incubated overnight at 4 °C with rabbit antisera anti-synthetic HAP2-peptide 2 diluted 1:2000 in TBS-T containing 1% of non-fat dried milk. Membranes were washed again and incubated with an anti-rabbit IgG conjugated with peroxidase (1:500) (Jackson ImmunoResearch) at room temperature for 1 h. After further washing with TBS-T, proteins of interest were detected by chemiluminescence by using ECL-Prime Western Blotting Detection Reagent (GE Healthcare, Chicago, USA). Pre-immune serum diluted 1:2000 was used as a negative control.

### Zygote formation blocking by anti-HAP2 antibodies

An eight-month-old splenectomized bovine was infested with 10,000 *B. bigemina*-infected *Rhipicephalus microplus* larvae. Twenty-nine days later when the infected bovine presented clinical signs and the parasitemia (determined by Giemsa-stained blood smear counts), raised more than 3%, whole blood was obtained from the jugular vein, defibrinated with glass beads and used for sexual stages induction using a previously described protocol [23] with modifications. Briefly, *B. bigemina* infected erythrocytes were cultured using induction medium and then monitored by Giemsa-stained smears each hour until zygote formation was confirmed at 36 h. At the same time, additional cultures were set up adding rabbit antiserum against two HAP2 peptides (pep2, pep3) at a 1:10 proportion. Each experiment was performed in triplicate and serum from rabbits immunized only with adjuvant was used as negative control.

When zygotes were detected in the normal induction cultures, all the cultures were centrifuged and the supernatant discarded, zygotes were recovered using a Percoll purification gradient as described before [23]. The cells were collected from the interface and washed three times with VYM buffer, after the last wash, the pellet was resuspended in 100 μl of PBS and smears were prepared and stained with Giemsa. Zygotes were counted across the smear surface (more than one hundred fields) and differentiated from later sexual stages by form as described previously [23] and percentages of zygote formation were calculated. All data were expressed as means percentage considering as a 100% all the cells counted including zygotes, extra and intraerythrocytic parasites. An ANOVA analysis was performed using the statistical software SPSS v.22 with a significance level of 0.05.

### Statistical analysis

All data for semiquantitative RT-PCR were expressed as the means ± SEM of three independent experiments in each case. The significance of the difference between means was determined by one-way ANOVA with Bonferroni *post-hoc* test using the software Graph Prism 5.0 with *F*_(5, 6)_ = 86.71. Comparisons were considered significant at *P* ≤ 0.0001. To analyze the inhibition of zygote formation, all data were expressed as means percentage considering as a 100% all the cells counted including zygotes, extra and intraerythrocytic parasites ± SEM of three replicas. A one-way ANOVA analysis was performed using the statistical software SPSS v.22 with *F*_(3, 8)_ = 69.574 with a significance level of *P* ≤ 0.05.

## Results

### *Babesia bigemina* HAP2 is highly conserved among strains

**Fig. 1:**
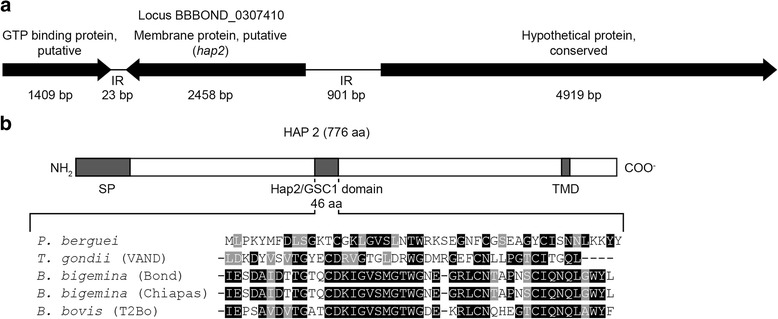
Localization and analysis of *B. bigemina hap2* gene. **a** Localization of *hap2* on chromosome III. The BLASTp analysis identified a sequence (XP_012769023.1) referred as "putative membrane protein of *B. bigemina*” in the GenBank, with the domain HAP2_GSC1 (pfam_pfam10699), in the locus BBBOND_0307410. **b** HAP2 scheme of Chiapas-México strain (KX768110), indicating in dark boxes the signal peptide (SP), the hap2/GSC1 domain and the transmembrane helix domain (TMD). The sequence of the HAP2-GSC1 domain of Chiapas-México strain was aligned with the following sequences: *Babesia bigemina* BBOND strain (XP_012769023.1), *Plasmodium berguei* (EU369602), *Toxoplasma gondii* VAND strain (KFH08353.1), *Babesia bovis* strain T2Bo strain (XP_001611806.1) by using Clustal Omega and edited with the BOXSHADE program. The black boxes show homologous sequences and the grey box denotes similar sequences

The HAP2 amino acid sequence of *P. berguei* was used to search for homologous genes in the *B. bigemina* genome. A *hap2* hypothetical gene was identified, with a size of 2458 bp located on chromosome III, on the complementary strand, in the BBOND_0307410 locus (Fig.1a).

The presence of a signal peptide in the first 70 aa of the protein was detected, as well as a large extracellular region of 666 aa and a 22-aa transmembrane helix at the carboxy(C)-terminus (Fig. 1b).

To examine whether the *hap2* gene is conserved in *B. bigemina* strains among geographically different regions, we sequenced the whole gene of Seed-Mexico (a vaccinal strain), Rondonia-Brazil and Kayseri-Turkey. The nucleotide sequence analysis of the strains (Chiapas-Mexico, Kayseri-Turkey Seed-Mexico and Rondonia-Brazil) showed the presence of four introns at the 5′ end, and one open reading frame encoding a protein of 778 aa for the Rondonia-Brazil strain and 776 aa for the rest of the strains. The protein codified by the *hap2* gene of the four strains shares the domain and a high level of identity from 97.82% between Rondonia-Brazil strain and BBOND strain reported in GenBank, to a 99.15% between Chiapas-México strain and Kayseri-Turkey strain. The C-terminus of the protein has a high level of basic amino acid residues, mainly Arg (Additional file 1: Figure S1).

A BLASTp analysis of the Chiapas-México strain showed a HAP2-GSC1 domain (pfam_pfam10699) of 46 aa. The multiple sequence alignment of Chiapas HAP2-GSC1 domain revealed a high identity among *Babesia* spp. showing a 100% of identity between the two strains of *B. bigemina* and a 67.39% with the domain of *B. bovis* (Fig. 1b) although the identity with other apicomplexans (*Plasmodium bergue*i and *Toxoplasma gondii*) was lower, 34.78 and 38.10%, respectively.

### HAP2 is expressed in sexual stages, but not in intraerythrocytic stages

**Fig. 2:**
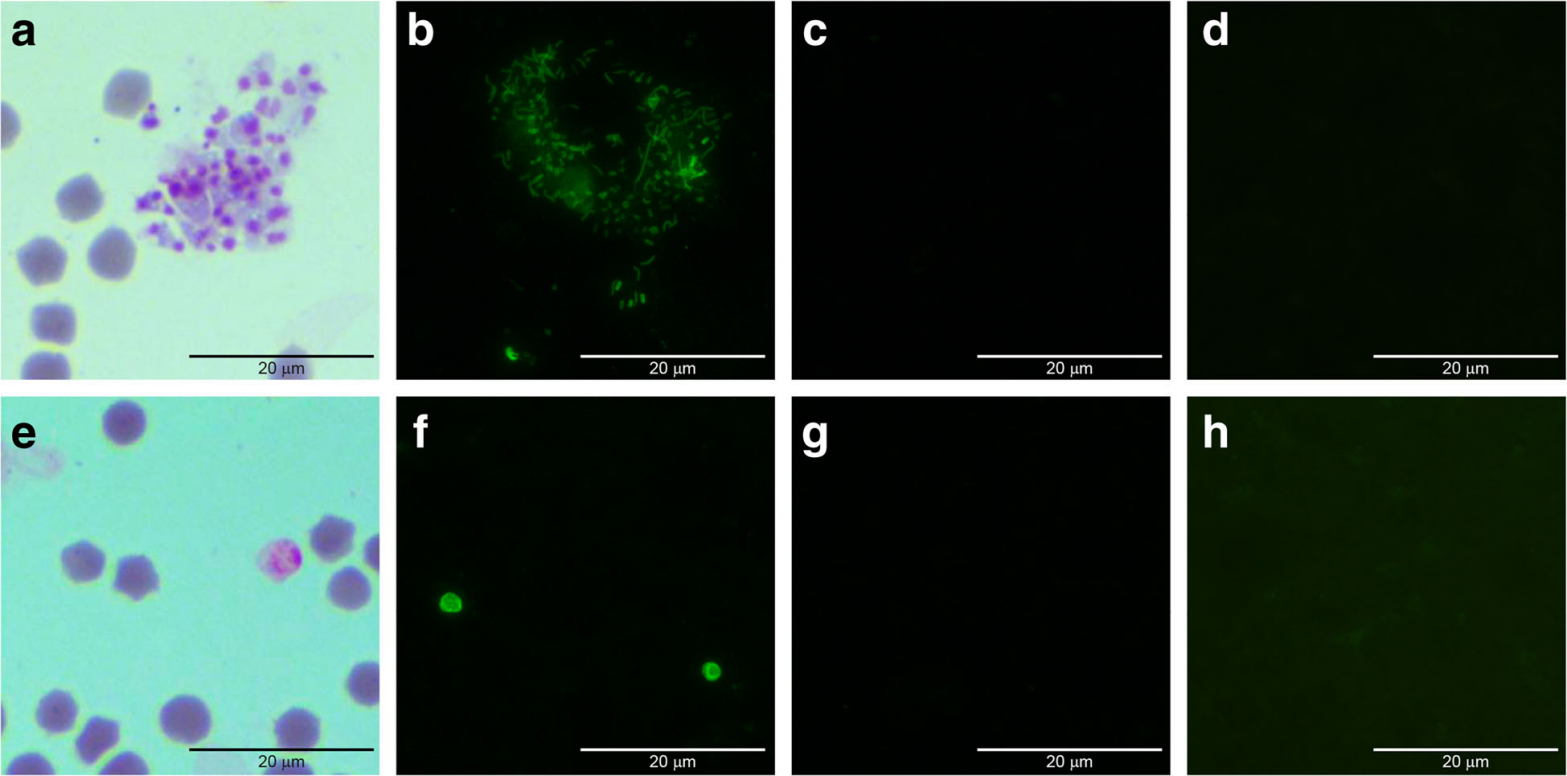
Analysis of HAP2 expression in sexual and asexual stages of *Babesia bigemina* by indirect immunofluorescence. *B. bigemina* sexual stages at 22 and 36 h post-induction, and asexual stages at 0 h were evaluated with antibodies against HAP2. **a-c** Sexual stages 22 h post-induction. **e-g** Sexual stages at 36 h post-induction. **d**, **h** Infected erythrocytes. **a**, **e** Giemsa stained sexual stages at 22 and 36 h post-induction, respectively. Sexual stages at 22 h (**b)**, or at 36 h post-induction **(f)**, as well as asexual, intraerythrocytic merozoites (**d**), were incubated with anti-HAP2 antiserum. Sexual stages at 22 h (**c)**, or at 36 h post-induction **(g)**, as well as asexual, intraerythrocytic merozoites **(h)** were incubated with pre-immune serum (1000×)

**Fig. 3:**
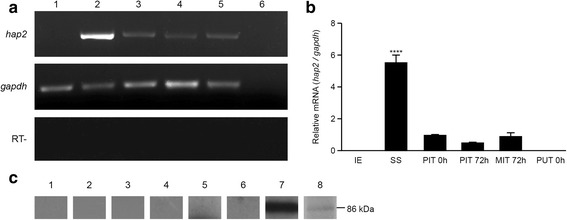
Expression of HAP2 in bovine and tick stages of *Babesia bigemina*. **a** RT-PCR of *hap2* and *gapdh* as a control. For both genes: Lane 1: infected erythrocytes; Lane 2: in vitro-induced sexual stages; Lane 3: pool of infected ticks at 0 h post-repletion; Lane 4: pool of infected ticks 72 h post-repletion; Lane 5: midgut of infected ticks 72 h post-repletion; Lane 6: pool of uninfected ticks at 0 h post-repletion. The lower panel corresponds to RT- controls of *hap2*. **b** Semiquantitative analysis of RT-PCR assays. Transcripts of *hap2* were quantified in relation to *gapdh* gene. Mean value ± SE of 2 quantifications is represented. The asterisks indicate the values significantly different from the control (ANOVA, *F*_(5, 6)_ = 86.71, *P* < 0.0001). **c**. Western blotting assays using total extracts of infected erythrocytes (Lanes 1 and 5), uninfected erythrocytes (Lanes 2 and 6), sexual stages (Lanes 3 and 7) and midgut of infected ticks at 72 h post-repletion (Lanes 4 and 8) were probed with anti-HAP2 antiserum (Lanes 5–8) or pre-immune serum (Lanes 1–4). The molecular mass marker is shown in kiloDaltons (kDa)

There is no report of this gene and its expression in any *Babesia* species so far. Interestingly, the anti-peptide antibody specific to HAP2 recognized the protein in sexual stages induced in vitro at 22 h post-induction (Fig. 2b) and in zygotes at 36 h post-induction (Fig. 2f). On the other hand, we found that the gene is expressed in sexual stages induced in vitro and stages of infected ticks, being significantly higher in sexual stages probably due to the purification process (Fig. 3a, Lane 2; 3b). We did not detect any level of expression in infected erythrocytes either in the mRNA or protein levels (Fig. 3a, Lane 1; 3c, Lane 5). The anti-HAP2 peptide antibody revealed a protein of apparent molecular weight of 86 kDa in sexual stages (Fig. 3c, Lane 7), and in midgut of infected ticks at 72 h post-repletion (Fig. 3c, Lane 8) the protein band is not observed in infected (Fig. 3c, Lane 5) or uninfected erythrocytes (Fig. 3c, Lane 6). The control serum did not show any positive signal (Fig. 3c, Lanes 1–4).

### Anti-HAP2 antibodies block zygote formation

**Fig. 4:**
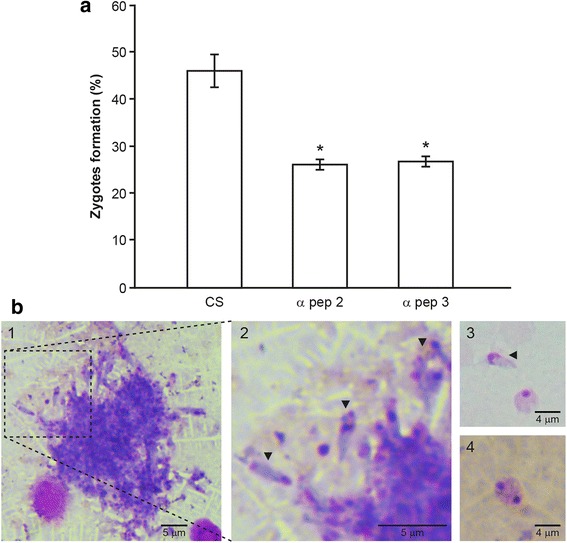
Anti-HAP2 antibodies block zygote formation. **a** The percentage of zygote formation was determined in cultures treated with antibodies against HAP2 immunogenic peptides 2 (α pep 2) and 3 (α pep 3), respectively. Serum from a rabbit immunized only with adjuvant was used as a control for zygote induction (CS); all data are expressed as a mean percentage considering as a total all the cells counted, including zygotes and extra and intraerythrocytic parasites. The asterisks indicate the values that are significantly different from the control (*P* < 0.05). **b** Representative images from zygote induction at 36 h. Sexual stages aggregate in Giemsa-stained smear prepared from the culture with anti-peptide 2 antibodies (1). Magnification of image **b**, black arrowheads show sexual stages (2). Zygotes induced with rabbit serum immunized only with adjuvant (3, 4). All smears were stained with Giemsa and observed at 100× magnification. *Scale-bars*: b1, 5 μm; b2, 5 μm; b3, 4 μm; b4, 4 μm

To explore if HAP2 of *B. bigemina* is playing a role during the sexual reproduction of the parasite, zygote formation was determined in vitro*,* and the comparison between cultures with anti-HAP2 antibodies and control was performed. As shown in Fig. 4a, the percentage of zygote formation in the culture with anti-HAP-2 peptide 2 and peptide 3 antibodies decreased to 26.34 and 26.70%, respectively. There were statistically significant differences among control cultures with adjuvant and cultures treated with serum from rabbits immunized with any of the HAP2 peptides (ANOVA analysis, *P* < 0.05).

Sexual stages were observed forming aggregates in cultures treated with anti-HAP2 antibodies Fig. 4b (Panels 1, 2), in comparison with the cultures with control serum where zygotes were appreciated as it is observed in Fig. 4b (Panel 3).

## Discussion

The identification and study of proteins with a potential role during fertilization in sexual reproduction is vital and has been shown to be considered as potential candidates for the development of transmission-blocking vaccines [16, 24]. Nevertheless, there is only one report about the proteins involved in sexual reproduction of *B. bigemina* [19].

This study describes the isolation and characterization of *hap*2 gene of *B. bigemina* which is only expressed in sexual stages induced in vitro and in infected ticks. Relevantly specific antibodies anti-HAP2 inhibited the zygote formation in vitro. The function of HAP2 in gamete fusion and fertilization is extensively documented from plants to unicellular eukaryotes [8–11, 25]. HAP2 is considered that was present in an ancestor common to all eukaryotes [13, 26] and has a structural and evolutionary relationship with Class II viral fusion proteins [5, 27].

Here, we demonstrated that the *B. bigemina* genome has a functional *hap2* gene which is highly conserved among geographically different strains. The amino acid sequences of the four *B. bigemina* strains have a conserved HAP2-GSC1 domain in the extracellular portion of the protein, a signal peptide and a transmembrane domain (TDM), which is consistent with the structure of HAP2 previously reported [12, 24]. HAP2 has a conserved structure with an extracellular region rich in cysteine containing a signal sequence and the HAP2-GCS1 (H/G) domain followed by a TDM domain and the cytoplasmic region with abundant positively charged amino acid residues. The N-terminus of the protein is sufficient for gamete fusion and the HAP2-GCS1 highly conserved domain is critical for fertilization in both plants and parasites [28]. The HAP2 ectodomain and TDM play an important role in protein trafficking to the cell surface, and the cytoplasmic domain has an important function in targeting the protein to the mating structure and regulating the fusion reaction [29].

HAP2 (GCS1) requires a C-terminus domain positively charged for the fusion of gametes [30]. Our study shows that HAP2 of *B. bigemina* has a C-terminus rich in basic amino acid arginine. In plants, the predominant basic amino acid is histidine while lysine and arginine are enriched in other species [10, 11, 26, 30]. It has been suggested that this C-terminal, positively charged domain of HAP2 could be crucial for the asymmetric fusion with a neighboring cell not expressing the protein [27], although the C-terminus function is still a controversial point in GCS1/HAP2 studies [31].

To investigate the expression and function of HAP2 of *B. bigemina*, an in vitro model for sexual stage development [23] was used, considering the technical restrictions to elucidate the molecular mechanisms during sexual stages development of *B. bigemina* within the vector ticks. The results presented here show that *hap2* gene is expressed in *B. bigemina* sexual stages induced in vitro and infected ticks. Interestingly, there is no expression of the gene in infected erythrocytes either in the mRNA or protein levels. Similar results were obtained in studies with *P. berguei* where *hap2* transcripts were not present in asexual erythrocytic stages [10]. Studies have shown that HAP2 is a sexual-stage-specific protein in *Plasmodium* [10, 12] being essential for sexual stages development and mosquito transmission in *P. berguei* [9].

Interestingly, we found that antibodies specific to HAP2, recognized the protein in both sexual stages and in zygotes induced in vitro. It is well known that HAP2 is a gamete-specific protein active in male gametes in *Chlamydomonas* and *Plasmodium* [10] and indispensable in both fusing gametes in *Tetrahymena thermophila* [32]. In *B. bigemina* sex-specific markers have not been reported yet, therefore is of great importance to further investigate if HAP2 is only expressed in male gametes.

We found that specific antibodies against HAP2 significantly decreased the zygote formation in vitro. Similarly, in *P. berguei* it has been shown that specific antibodies anti-HAP2 significantly inhibited ookinete formation in vitro*,* suggesting that anti-HAP2 antibodies have a transmission blocking effect during fertilization or during the transformation of the zygote to the ookinete [10].

In summary, our findings indicate that HAP2 is a specific sexual stage protein in *B. bigemina* that might be playing a key function during sexual development of the parasite, hence the relevance to further study this protein as a possible candidate for a transmission-blocking vaccine.

## Conclusion

This paper shows that *hap2* is a functional gene of *B. bigemina* highly conserved among different strains and that it is expressed in sexual stages of the parasite and within the vector tick. Besides specific antibodies, anti-HAP2 were able to inhibit the zygote formation in vitro. Altogether, our results suggest that *B. bigemina* HAP2 could be playing the same role as it has been reported in other apicomplexan species and this protein should shortly be considered as a potential candidate for a transmission-blocking vaccine against *Babesia* spp. Moreover, more work is needed to investigate whether HAP2 is a male-specific protein in *Babesia* and its function in the parasite biology.

## Additional file

Additional file 1: Figure S1. Alignment of the HAP2 putative amino acid sequences. a Comparison of the amino-acid sequences of HAP2 in several *B. bigemina* strains from different geographical regions; sequence alignment was performed in Clustal Omega at http://www.ebi.ac.uk/Tools/msa/clustalo/. Shadow background indicates the area of HAP2/GCS domain. The sequences corresponding to the synthetic peptides (pep2 and pep3) are shown by an upper line. The TDM is shown in darker grey, and the basic amino acids residues are shown at C-terminus. (PDF 110 kb)
