# Supplementary material for: Hap2, a novel gene in Babesia bigemina is expressed in tick stages, and specific antibodies block zygote formation
Source: Parasit Vectors. 2017 Nov 13;10:568. doi: 10.1186/s13071-017-2510-0 (PMC5683354; doi:10.1186/s13071-017-2510-0)
Supplement: Additional file 1: Figure S1. — Alignment of the HAP2 putative amino acid sequences. a Comparison of the amino-acid sequences of HAP2 in several B. bigemina strains from different geographical regions; sequence alignment was performed in Clustal Omega at http://www.ebi.ac.uk/Tools/msa/clustalo/. Shadow background indicates the area of HAP2/GCS domain. The sequences corresponding to the synthetic peptides (pep2 and pep3) are shown by an upper line. The TDM is shown in darker grey, and the basic amino acids residues are shown at C-terminus. (PDF 110 kb) [file 13071_2017_2510_MOESM1_ESM.pdf]

```
                                                                          pep2
XM_012913569.1    MTHAVLNRSYKSPLRGAACLLALLIGAAVRPASAVIISPVRQCIDKGGRSVAEGDC-WRS
Ro-Brazil         MTHAVLNRSNKSPLRVAACLLALLIGAAVRPASAVIISPVRQCIDKGGRSVAEGDCEWRS
Seed-México       MTHAVLNRSYKSPLRGAACLLALLIGAAVRPASAVIISPVRQCIGKGGRSVAEGDCEWRS
Chi-México        MTHAVLNRSYKSPLRVAACLLALLIGAAVRPASAVIISPVRQCIDKGGRSVAEGDCEWRS
Kay-Turkey        MTHAVLNRSYKSPLRVAACLLALLIGAAVRPASAVIISPVRQCIDKGGRSVAEGDCEWRS
                  ********* ***** ***************************** *********** ***
                                          pep3
XM_012913569.1    HTNIDVKGGEETASYVLRRKDKPNSGLYIHIQTSLTTLSYELEYIMDVPSQYREHN-SVD
Ro-Brazil         HTNIDVKGGEETASYVLRRKDKPNSGLYIHIQTSLTTLSYELEYIMDVPSQYREHNRSVD
Seed-México       HTNIDVKGGEETASYVLRRKDKPNSGLYIHIQTSLTTLSYELEYIMDVPSQYREHNRSVD
Chi-México        HTNIDVKGGEETASYVLRRKDKPNSGLYIHIQTSLTTLSYELEYIMDVPSQYREHNRSVD
Kay-Turkey        HTNIDVKGGEETASYVLRRKDKPNSGLYIHIQTSFTTLSYELEYIMDVPSQYREHNRSVD
                  **********************************:********************* ***

XM_012913569.1    YSAMTGGCNNCDALELEICSKPDALPPSIQESFNNKACCVCGVNVTDYSPRADFSC-GFG
Ro-Brazil         YSAMTGGCNNCDALDLEICSKPDALPPSIQDSFNNKACCVCGKNVTDYSPRADFSCTGFG
Seed-México       YSAMTGGCNNCDALDLEICSKPDALPPSIQDSFNNKACCVCGKNVTDYSPRADFSCTGFG
Chi-México        YSAMTGGCNNCDALDLEICSKPDALPPSIQDSFNNKACCVCGKNVTDYSPRADFSCTGFG
Kay-Turkey        YSAMTGGCNNCDALDLEICSKPDALPPSIQDSFNNKACCVCGKNVTDYSPRADFSCTGFG
                  **************:***************:*********** ************* ***

XM_012913569.1    AAFMHGKCVSMSCLEVVGPWYSIFKPAYPPTIHRRVFVDVYAFDGDAGVVPDVAKN-YYT
Ro-Brazil         AAFMHGKCVSMSCLEVVGPWYSIFKPAYPPTIHRRVFVDVYAFDGDAGVVPDVAKNGYYT
Seed-México       AAFMHGKCVSMSCLEVVGPWYSIFKPAYPPTIHRRVFVDVYAFDGDAGVVPDVAKNGYYT
Chi-México        AAFMHGKCVSMSCLEVVGPWYSIFKPAYPPTIHRRVFVDVYAFDGDAGVVPDVAKNGYYT
Kay-Turkey        AAFMHGKCVSMSCLEVVGPWYSIFKPAYPPTIHRRVFVDVYAFDGDAGVVPDVAKNGYYT
                  ******************************************************** ***

XM_012913569.1    SDDADETRYLKEAVYKDKHLKATLSAEKQAVKNEELDVTLTIITQQWLDGNAPQKM-KFV
Ro-Brazil         SDDADETRYLKEAVYKDKHLKATLSAEKQAVKNEELDVTLTIITQQWLDGNAPQKMDKFV
Seed-México       SDDADETRYLKEAVYKDKHLKATLSAEKQAVKNEELDVTLTIITQQWLDGNAPQKMDKFV
Chi-México        SDDADETRYLKEAVYKDKHLKATLSAEKQAVKNEELDVTLTIITQQWLDGNAPQKMDKFV
Kay-Turkey        SDDADETRYLKEAVYKDKHLKATLSAEKQAVKNEELDVTLTIITQQWLDGNAPQKMDKFV
                  ******************************************************** ***

XM_012913569.1    AVPSWPETDKTVQGSSRKYNCDQEGRDPHECEHDDVRCRMEKCALNVRTIESDAID-TGT
Ro-Brazil         AVPSWPETDKTVQGSSRKYNCDQEGRDPHDCEHDDVRCRMEKCALNVRTIESDAIDTTGT
Seed-México       AVPSWPETDKTVQGSSRKYNCDQEGRDPHDCEHDDVRCRMEKCALNVRTIESDAIDTTGT
Chi-México        AVPSWPETDKTVQGSSRKYNCDQEGRDPHDCEHDDVRCRMEKCALNVRTIESDAIDTTGT
Kay-Turkey        AVPSWPETDKTVQGSSRKYNCDQEGRDPHDCEHDDVRCRMEKCALNVRTIESDAIDTTGT
                  *****************************:************************** ***
                       HAP2-GSC1
XM_012913569.1    QCDKIGVSMGTWGNEGRLCNTAPNSCIQNQLGWYLSERKER------------ALLPKLY
Ro-Brazil         QCDKIGVSMGTWGNEGRLCNTAPNSCIQNQLGWYLSERKERVGSGHERPHCVQALLPKLY
Seed-México       QCDKIGVSMGTWGNEGRLCNTAPNSCIQNQLGWYLSERKERVGSGHERPHGVQALLPKLY
Chi-México        QCDKIGVSMGTWGNEGRLCNTAPNSCIQNQLGWYLSERKERVGSGLERPHCVQALLPKLY
Kay-Turkey        QCDKIGVSMGTWGNEGRLCNTAPNSCIQNQLGWYLSERKERVGSGHERPHCVQALLPKLY
                  *****************************************            *******

XM_012913569.1    GVQPMLAR-EVRKSKQAAAMEEPEDLAEESRSP-TAKSAWRGPATYGAEDEGEEDE----
Ro-Brazil         GVQPMLARREVRKSKQAAAMEEPESLAEESRSSSMSKSAWRGPATYGAED---------E
Seed-México       GVQPMLARREVRKSKQAAAMEEPENLAEESRTS-TAKSAWRGPATYGAEDEDEGEEDEDE
Chi-México        GVQPMLARREVRKSKQAAAMEEPESLAEESRTS-TAKSAWRGPATYGAEDEDEGEEDEDE
Kay-Turkey        GVQPMLARREVRKSKQAAAMEEPESLAEESRSS-TAKSAWRGPATYGAEDEDEGEEDEDE
                  ******** ***************.******:   :**************

XM_012913569.1    -DEDEDQEPDEWHD-TYVHAVAYSIAKADTSRIEINTFDATVTQIIAEAVGFIVSATMDG
Ro-Brazil         GEEDEDQEPDEWHDDTYVHAVAYSIAKADTSRIEINTFDATVTQIIAEAVGFIVSATMDG
Seed-México       GEEDEDEEPDEWHDDTYVHAVAYSIAKADTSRIEINTFDATVTQIIAEAVGFIVSATMDG
Chi-México        GEEDEDEEPDEWHDDTYVHAVAYSIAKADTSRIEINTFDATVTQIIAEAVGFIVSATMDG
Kay-Turkey        GEEDEDEEPDEWHDDTYVHAVAYSIAKADTSRIEINTFDATVTQIIAEAVGFIVSATMDG
                   :****:******* *********************************************

XM_012913569.1    PCKVASHEACTMKI-TKNAGKIRAKFSHRIQCYEAEKPHTSPAASSEEQSVQVEPNSTTS
Ro-Brazil         PCKVASHEACTMKIVTKNAGKIRAKFSHRIQCYEAEKPHTSPAASSEEQSVQVEPNSTTS
Seed-México       PCKVASHQACTMKIVTKNAGKIRAKFSHRIQCYEAEKPHTSPAASSEEQSVQVEPNSTTS
Chi-México        PCKVASHEACTMKIVTKNAGKIRAKFSHRIQCYEAEKPHTSPAASSEEQSVQVEPNSTTS
Kay-Turkey        PCKVASHQACTMKIVTKNAGKIRAKFSHRIQCYEAEKPHTSPAASSEEQSVQVEPNSTTS
                  *******:****** *********************************************

XM_012913569.1    SVVPIKILSGEGSD-LECEVQLYSSTVSLLETFVVKASLATPTITVGPDVRSFDKFTDTS
Ro-Brazil         SVVPIKILSGEGSDKLECEVQLYSSTVSLLETFVVKASLATPTITVGPDVRSFDKFTDTS
Seed-México       SVVPIKILSGEGSDKLECEVQLYSSTVSLLETFVVKASLATPTITVGPDVRSFDKFTDTS
Chi-México        SVVPIKILSGEGSDKLECEVQLYSSTVSLLETFVVKASLATPTITVGPDVRSFDKFTDTS
Kay-Turkey        SVVPIKILSGEGSDKLECEVQLYSSTVSLLETFVVKASLATPTITVGPDVRSFDKFTDTS
                  ************** *********************************************
                                                                       TDM
XM_012913569.1    KEDHALKIGAMLS-DMCTCNAGEVGCFFRNFGKCMRHAFNKYYTWFVLGITGLSFLALLP
Ro-Brazil         KEDHELKIGAMLSDNMCTCNAGEVGCFFRNFGKCMRHAFNKYYTWFVLGITGLSFLALLP
Seed-México       KEDHELKIGAMLSDNMCTCNAGEVGCFFRNFGKCMRHAFNKYYTWFVLGITGLSFLALLP
Chi-México        KEDHELKIGAMLSDNMCTCNAGEVGCFFRNFGKCMRHAFNKYYTWFVLGITGLSFLALLP
Kay-Turkey        KEDHELKIGAMLSDNMCTCNAGEVGCFFRNFGKCMRHAFNKYYTWFVLGITGLSFLALLP
                  **** ******** :*********************************************

XM_012913569.1    VFIPLARVVFGKISEGRARARIRREAAQEIERIRREFHHDEERYGRPEFRSRHSDEV
Ro-Brazil         VFIPLARVVFGKISEGRARARIRREAAQEIERIRREIHHDEERYGRPEFRSRHSDEV
Seed-México       VFIPLARVVFGKISEGRARARIRREAAQEIERIRREIHHDEERYGRPEFRSRHSDEV
Chi-México        VFIPLARVVFGKISEGRARARVRREAAQEIERIRREIHHDEERYGRPEFRSRHSDEV
Kay-Turkey        VFIPLARVVFGKISEGRARARIRREAAQEIERIRREIHHDEERYGRPEFRSRHSDEV
                  *********************:**************:********************
```
